# Student Perceptions of Interprofessional Education Innovation to Address Pain Management and Substance Use: A Qualitative Descriptive Analysis

**DOI:** 10.1111/jan.70406

**Published:** 2026-01-26

**Authors:** Rie Kobayashi, Jennifer C. Miller, Connie M. Remsberg, Skye McKennon, Ross J. Bindler, Dawn E. DeWitt, Marian Wilson

**Affiliations:** ^1^ School of Social Work Eastern Washington University Cheney Washington USA; ^2^ College of Pharmacy and Pharmaceutical Sciences Washington State University Spokane Washington USA; ^3^ Elson S. Floyd College of Medicine Washington State University Spokane Washington USA; ^4^ College of Nursing Washington State University Spokane Washington USA

**Keywords:** interprofessional education, qualitative evaluation, simulation, standardised patient, student perspectives

## Abstract

**Aims:**

The aim of this study was to understand student perspectives regarding an interprofessional education (IPE) innovation using a single standardised patient (SP) in a large‐group setting for a pain management and substance use simulation.

**Design:**

A qualitative descriptive design was used.

**Method:**

Students representing eight health science programs from four universities were invited to participate in a simulation‐based IPE program blending asynchronous and synchronous learning. DNP students were one of the largest professions represented (*n* = 92, 30%) along with students enrolled in Doctor of Pharmacy (*n* = 111) and Doctor of Medicine (*n* = 69) programs. Students were invited to complete a post‐activity survey asking what parts of the IPE activity were most valued and what could be improved. Student responses were themed using a qualitative descriptive approach with inductive coding and constant comparison.

**Results:**

Of 304 participating students, 155 (51%) responded to one or both open‐ended questions. Respondents highly valued interprofessional team diversity. Responses highlighted the importance of: (1) using simulation with student teams to foster active learning, (2) student preparation using relevant curricular resources and (3) grounding interprofessional collaboration activities in student engagement and professional respect.

**Conclusion:**

Findings confirmed that a cost‐effective IPE activity using one SP within deliberately planned interprofessional activities can be engaging and meaningful. Students valued team‐based collaboration across the disciplines of nursing, pharmacy and medicine.

**Impact:**

Educators gathered evidence on the merits of a replicable, cost‐effective IPE structure intended to expand team‐based simulation learning opportunities. High‐priority public health topics such as pain and substance use require multidisciplinary, integrative care to maximise health outcomes. To better prepare nurses and their health science collaborators, novel pedagogy in IPE may optimise student learning experiences.

**Reporting Method:**

We followed the Standards for Reporting Qualitative Research (SRQR).

**Patient or Public Contribution:**

Health sciences faculty served as facilitators in the IPE sessions. Facilitators were provided 1 h of training and observed student team breakout rooms to ensure that students were engaged and understood the assigned task. They provided feedback to session leaders after the sessions.

**Summary**

- What does this paper contribute to the wider global clinical community?
  ○ Insight into cost‐effective approaches that educators can use to actively engage health science professions, including advanced nursing students, in authentic interprofessional learning activities.
  ○ Providing focused curricular resources that prepare diverse student learners, including advanced nursing students, is critical.

## 1 Introduction

Substance use disorders (SUDs) are global public health concerns. Drug use disorders affect an estimated 64 million people globally with only 1 in 11 receiving treatment (United Nations Office on Drugs and Crime 2024). Higher rates are reported in the U.S., with a national survey from the Substance Abuse and Mental Health Services Administration (SAMHSA), estimating that 48.4 million Americans or 16.8% of the population aged 12 or older had a substance use disorder in 2024 including 28.2 million experiencing alcohol use disorder (AUD) (SAMHSA 2024). SUD treatment is further complicated, as many patients with chronic pain have a comorbid SUD (Morasco et al. 2011; Dowell et al. 2022). Those with comorbid pain and SUD are at increased risk of receiving inadequate pain treatment (Dowell et al. 2022).

To address this global public health crisis, an interprofessional approach is needed. In 2021, the National Academy of Medicine, in response to the U.S. opioid crisis, released a special publication emphasising the need to ‘recognize that interprofessional care is essential to manage both pain management and SUDs’ (Chappell et al. 2021). The publication also identified critical gaps in health science education on pain management and substance use (Chappell et al. 2021). To address these gaps, it is imperative that institutions provide interprofessional education (IPE) experiences that bring together students from multiple disciplines to discuss SUD. Not only should these IPE experiences highlight the assessment and treatment of pain and potential SUD, but also the importance of interprofessional collaboration to deliver holistic care as highlighted in SUD core curriculum recommendations from SAMHSA (S.A.M.H.S. Administration 2024).

Several reports in the literature describe positive outcomes associated with IPE activities addressing SUD‐related topics (Muzyk, Smothers, et al. 2020; Bishop et al. 2015; Fishman et al. 2020; Muzyk, Mullan, et al. 2020; Muzyk et al. 2019; Hager et al. 2018; Langford et al. 2020; Rotz et al. 2021; Monteiro et al. 2017). Most of these IPE activities utilise case scenarios and small group discussions of interprofessional student teams. Outside of our group's efforts, only one published study specifically focuses on SUD and incorporates interactions with standardised patients (SPs), paid actors who portray a patient case (Monteiro et al. 2017). In that study, teams of health professional students interviewed an in‐person SP directly, and not as part of a large group.

Although IPE activities with SPs provide students with relevant experiences, simulations using SPs are often logistically challenging and expensive. This can create significant barriers to implementing authentic IPE experiences. Interestingly, limited information is available about the effectiveness of creative and resource‐efficient approaches for using SPs within the IPE framework. Moreover, there are no published studies to our knowledge about students' perceptions around using a single SP in a large group IPE setting as described herein.

## 2 Background/Educational Approach

Since 2018, our interprofessional team, representing medicine, nursing, pharmacy and social work programs, has developed and offered an annual two‐part, longitudinal IPE training focused on pain and substance use to health profession students across the U.S. Pacific Northwest (Wilson et al. 2021; Remsberg et al. 2023; Wilson et al. 2023). The success of our pilot in 2019 (Wilson et al. 2021) led to the expansion of the training to include SP encounters where students follow changes in pain and substance use of a SP over time. Pairing two separate SP encounters, a month apart, with interprofessional team discussions provides a ‘low stakes,’ safe environment where common assumptions and beliefs regarding the stigmatised SUD and chronic pain population can be challenged. Because of funding and resource limitations, incorporation of SPs into the two virtual, IPE trainings required creativity and ultimately took different forms to train several hundred students per year. In the first encounter, each student team interacts with their own individual SP, while in the second follow‐up encounter, one SP interacts with multiple student teams allowing for a SP fee reduction of approximately $1000 per session. Descriptions of the first and follow‐up encounters along with results of pre‐ and post‐changes in knowledge and confidence have been previously published (Remsberg et al. 2023; Wilson et al. 2023).

Despite mandates for IPE within nursing and other health professions that progress beyond siloed training practices, it is widely recognised that research is lacking that can expand understanding about how IPE serves to prepare students for collaborative practice (Bianchi et al. 2021; O'Connor 2018). To expand on our understanding of key features that make IPE activities successful, this paper focuses on the IPE training experience from student perspectives by exploring what parts were most valued and suggestions for improving the second, follow‐up encounter that utilises only one SP. Our work fills a gap, as there is a lack of studies that specifically ask students about the usefulness of a single SP in a large‐group setting. This is pertinent in times of budgetary reductions to ensure that cost‐reduction decisions do not negatively affect students' learning opportunities, especially when training large groups of students from multiple professions, using one SP.

As previously described, the follow‐up IPE activity involved a blended approach of asynchronous and synchronous learning (Wilson et al. 2023). Prior to the virtual synchronous session, students completed a 60–90‐min asynchronous module that included background on the patient case, an introduction to Screening, Brief Intervention and Referral to Treatment (SBIRT), pharmacotherapy for AUD and Opioid Use Disorder (OUD) and additional references (Bindler et al. 2025). The 110‐min, virtual synchronous session via the Zoom videoconferencing platform consisted of the following:

1. Large group introduction and session agenda;
2. Initial student‐led breakout (student teams introduce themselves, assign roles and review the patient case);
3. Large group session (a pre‐recorded telehealth video of this patient with a primary care clinician using SBIRT is shown);
4. Question‐and‐answer (Q & A) session (each team's spokesperson enters questions via the Zoom chat, a faculty facilitator verbally asks a single SP the submitted questions);
5. Second student‐led breakout (student teams prioritise the patient's problems and formulate a holistic treatment plan);
6. Large group debrief (a faculty facilitator summarises the key points of the case, and each team's spokesperson shares their problem list and treatment options via the Zoom chat).

The IPE activity is freely available for use and can be viewed in full along with all other course materials by submitting a request to the authors at https://spokane.wsu.edu/opioid‐education/.

## 3 The Study

A qualitative descriptive approach was employed to explore students' perceptions and experiences of the IPE training and ways to improve future IPE sessions. Because the training involved a change from previous IPE opportunities to interact with a SP in a small group setting and instead shared a single SP in a large group interview, the researchers wanted to understand students' experiences and ensure that their preferences were captured. At the conclusion of the IPE session, the session facilitator provided a link inviting students to complete a voluntary, anonymous quality‐improvement survey hosted in the primary university's Research Electronic Data Capture (REDCap). The survey included two open‐ended questions:

1. What part of the IPE curriculum did you find most valuable?
2. How could the IPE curriculum be improved?

While there was no specific timeline to complete the survey, most students completed the survey following sign‐off from their IPE session. The survey link was also provided to students in a brief ‘Thank You’ email following the session. All responses included in this analysis were completed within 6 weeks of the first IPE session.

## 4 Methods

### 4.1 Design

We adopted a qualitative descriptive research method based on the objective of obtaining straightforward descriptions of student experiences (Doyle et al. 2020). Such pragmatic designs are ideal for studies that do not require a deeply theoretical context, and data is intended to be used for quality improvement purposes (Doyle et al. 2020). We set out to explore student perceptions attributed to the IPE activity and contextual aspects within the multidisciplinary learning environment. We followed the Standards for Reporting Qualitative Research (SRQR) (O'Brien et al. 2014).

### 4.2 Study Setting and Recruitment

Students from eight health professions programs at four educational institutions participated in one of the four IPE activity offerings as part of their assigned coursework. Each scheduled session included slightly under 100 students (Range: 69–81 students). The data used for analysis was provided from voluntary post‐activity surveys.

### 4.3 Inclusion and/or Exclusion Criteria

All student data was included from completed post‐activity surveys with no exclusions.

### 4.4 Data Collection

All data for the primary research questions was captured within the university's REDCap system. Demographic data was limited to respondents' program of study. After all responses were submitted, a spreadsheet export of all valid responses to the two open‐ended questions from REDCap was created.

### 4.5 Data Analysis

Descriptive statistics were used to summarise student characteristics. Content analysis was used to analyse qualitative data (Doyle et al. 2020). Three analysis team members, including the first and second authors, independently read a subset of text responses, identified concepts and relationships from each response and recorded potential categorical codes. After the first subset of data was independently reviewed, the analysis team met and collaboratively created a list of potential categorical codes individually identified. Based on the discussion, a coding template was created. The analysis team applied the template to the remaining subsets independently and met several times as a team to discuss individual review of the data and coding. In doing so, the analysis team compared codes and interpretations within and across transcripts and discussed any discrepancies until consensus was reached. These discussions often resulted in consolidating overlapping codes and subcategories, developing new codes and refining the codebook. This approach helped the analysis team develop a more contextual understanding of the data and identify emerging themes. The coding and preliminary findings were presented to the rest of the IPE team for feedback. The first and second authors then re‐reviewed the coded data and revisited identified emerging themes independently and together several times to finalise analysis.

### 4.6 Ethical Considerations

The primary university's Human Research Protection Program certified this study as not a ‘Human Subjects Study’ and waived the need for Institutional Review Board approval. Students were provided with instructions assuring them of anonymity and that responses would not affect grades.

### 4.7 Rigour and Reflexivity

To ensure the rigour of the research process, data were reviewed independently and credibility was enhanced by using multiple members of the research team to review the coding and resulting themes. The coding team used inductive coding and constant comparison to ensure consistency and validity in coding and analysis. Trustworthiness was enhanced by keeping records of the raw data and sharing decisions made using an audit trail to document the analysis process.

## 5 Findings

### 5.1 Characteristics of Participants

The IPE activity involved 304 students from eight disciplines/programs: (1) pharmacy (Doctor of Pharmacy, PharmD) (*n* = 111, 36.5%), (2) nursing (Doctor of Nursing Practice, DNP) (*n* = 92, 30.2%), (3) medicine (Doctor of Medicine, MD) (*n* = 69, 22.7%), (4) athletic training program (Bachelor of Science) (*n* = 14, 4.6%), (5) social work (Bachelor of Arts in Social Work, BASW) (*n* = 7, 2.3%), (6) addiction studies (Associate in Arts) (*n* = 5, 1.6%) and (7) other (Ph.D. in Prevention Science; Master's in Nursing), (*n* = 6, 2%).

A total of 155 student IPE activity participants submitted responses to one or both open‐text questions on the post‐session survey (51% response rate). Pharmacy students responded the most (*n* = 58; 37.4% of all responses received), followed by nursing (*n* = 52; 33.5% [DNP = 50; 32.3%; MSN = 2; 1.3%]) and medicine (*n* = 32; 20.6%). Fewer responses came from athletic training (*n* = 6; 3.9%), social work (*n* = 3; 1.9%), addiction studies (*n* = 3; 1.9%) and prevention science PhD (*n* = 1; 0.6%) students. To ensure the anonymity of students completing the post‐activity feedback survey, only their educational program was collected. Demographic information such as sex, race, highest education level, etc. of the 304 students who attended the IPE activity has been published elsewhere (Wilson et al. 2023) and was collected as part of the live session registration and was not connected to the post‐IPE survey.

### 5.2 Themes

Three major themes emerged from the data. Specifically, students commented upon: (1) session format, (2) curriculum and (3) student teams. Identified themes, with subcategories, are presented below.

#### 5.2.1 Session Format

The major focus of the session format theme was the importance of participant engagement and active learning. Comments highlighted interprofessional student collaboration, activity structure and the organisation and logistics of the IPE session.

##### 5.2.1.1 Interprofessional Student Collaboration

Interprofessional collaborative opportunities were the aspect reported most frequently as valued (*n* = 80). The benefits of interprofessional collaboration were reported by most professions. Students noted that interprofessional participation facilitated a stronger learning experience, broadened perspectives and led to gaining/sharing discipline‐specific information. These exemplars reflect what students valued the most about the IPE session:

I enjoyed the grouping of people. We had a diverse group, and this helped with collaboration and a stronger learning experience. (DNP student)

I also liked collaborating with a broad base of healthcare professionals. It's imperative to take an interdisciplinary approach as it provides the opportunity to think outside of our disciplines and learn from another approach. (BASW student)

The ability to work with other professionals and discuss what role they played in the patient [sic] care. Each team member took time to discuss treatment modalities and other profession‐specific information to the others, which was very valuable and appreciated. (Pharmacy student)

I love interaction with other health professions. Going over the different medications was helpful (thank you pharmacists for your help). Assembling a treatment plan was a good exercise because it's what we will actually be doing. (Medical student)

##### 5.2.1.2 Small and Large Group Collaboration

Students recognised the use of both large group interactions and two small group breakouts as valuable parts of the session format (*n* = 39). The first breakout session focused on team member introductions, identifying team roles, discussing the patient case and preparing a list of questions for the SP. The second breakout focused on creating and submitting a holistic treatment plan. Large group interactions included an overview of the learning activity, a patient‐clinician video demonstrating SBIRT principles, follow‐up questions from the teams to the SP and a debrief. Learners commented that the strategic use of small and large group interactions strengthened their learning and enhanced engagement.

I liked that we could break into small groups to prepare for the first large work session, so we had an idea of what we wanted to specifically look for in the interview. (DNP student)

One pharmacy student commented, ‘the larger group interview was nice because it had more professions working together.’ Thus, the large group interview with the SP likely remedied a lack of diverse professions represented in some of the small group interactions and provided more interprofessional perspectives.

##### 5.2.1.3 Organisation and Time

The importance of well‐organised interactive activities and thoughtful allocation of time was highlighted in the student comments regarding recommended changes for improving the IPE session (*n* = 17). Students had varying thoughts and creative ideas about how to allocate the synchronous IPE session time and re‐organise the IPE session format. These comments reflect their desire to spend more time working on the patient case itself.

I wish we spent a bit less time on the intro and more time on the treatment plan brainstorm! We were trying to address all aspects of his care (depression, sleep, pain, SUD) and I don't think there was enough time to do that level of care. Plenty of time to discuss SUD medications though which was the focus! (Medical student).

Several students recommended a longer duration (a two‐day session) for this IPE session and more frequent IPE training sessions.

##### 5.2.1.4 Application of Discipline‐Specific Knowledge and Skills

Participants valued the opportunity to apply profession‐specific knowledge and skills (*n* = 12). The development of an interprofessional, holistic treatment plan for the SP was instrumental in assisting students to contextualise content, create solutions for identified problems experienced by the SP and team and examine the rationale behind decision making associated with treatment plans.

Working in small group [sic] to complete a patient treatment plan allowing me to learn from others' perspective and rationale for decision making. (DNP student)

Making a treatment plan with other students got us researching and discussing options, and it helped me to contextualize content. (Medical student)

Using our respective knowledge from our schooling to create solutions. (Pharmacy student)

##### 5.2.1.5 Utilisation of SP Actor

The utilisation of a standardised patient (SP) in the IPE session was mentioned by multiple students (*n* = 12). Students first viewed a recorded physician‐patient interaction video, demonstrating best practices and then participated in the live, interactive, large‐group SP interview. Throughout the duration of the large‐group SP interview, students were invited to submit questions they wanted to ask the SP using the Zoom chat function. The lead facilitator then asked the questions using motivational interviewing principles and non‐stigmatising language on behalf of the students, while simulating a team‐based healthcare appointment. The role of the SP was to provide a realistic and consistent environment for learners to practice and refine their clinical and communication skills. The SP completed training with our simulation centre faculty and lead facilitators and engaged in practice sessions with the lead facilitators. The live SP interview was noted as helpful for allowing students to ask additional questions of the SP and for getting all students on the same page.

I found the question‐asking portion after the video most valuable as it was able to get everyone caught up regarding the patient's condition and answer some burning questions that students had. (Pharmacy student)

On the other hand, students also found the live SP interview challenging. Participants offered recommendations such as streamlining the SP question and answer session, as well as discussing a treatment plan with and getting feedback from SP for improving the SP interview.

I feel like there should be a better way to organize/have groups submit questions during the large group interactive session. It felt a bit chaotic with so many questions being entered into the chat all at once. (Pharmacy student)

If anything, asking the additional questions could be more streamlined? I thought the facilitator did a good job trying to ask everything and grouping questions as best she could though. (Medical student)

[It would be helpful to…] talk to the patient and have his opinion about the treatment plan. (BASW student)

#### 5.2.2 Curriculum Development

Curriculum development was another major theme that emerged from the data. Subcategories included: (1) relevant content, (2) preparatory materials/resources and (3) modelling skills, as key ingredients for enhancing learning and IPE.

##### 5.2.2.1 Relevant Content

Students commented on the importance, individually and professionally, of seeing the relevance and value of content included in the IPE session (*n* = 31). They noted the value of specific content, that is, specialised knowledge, such as how to taper off opioids, symptoms to look for in opioid withdrawal, pharmacotherapy for AUD/OUD and holistic approaches to treating patients. Curricular enhancement ideas provided by students included: increasing focus on motivational interviewing techniques; prescribing and monitoring of medications for AUD/OUD, medication titration and alternative pain treatments; addressing addiction and suicide more directly; and better incorporation of physical exercise as therapy.

##### 5.2.2.2 Preparatory Materials/Resources

An asynchronous preparatory learning activity (< 90 min) was designed by the interprofessional faculty team to provide students from diverse professions with a common foundation for the synchronous IPE session. Materials included a synchronous session instruction guide, the patient case and information on SBIRT and pharmacotherapy treatment for AUD and OUD. External links to additional references were included to support students' learning about other content as needed, including Centers for Disease Control and Prevention SUD treatment links and other national websites for substance use treatments in pregnancy, suicide assessment and stigma awareness. Student comments recognised the value of this preparation (*n* = 25).

I found the extensive background information for the case study helpful. (MSN student)

Offering resources for future practice, as this could be an issue we see tomorrow or in years, having resources is a good refresher. (DNP student)

##### 5.2.2.3 Modelling Skills

Deliberate modelling of clinician behaviours and skills, as an educational feature, was valued by students (*n* = 18). A video modelling physician‐patient interaction during the synchronous session helped students see the SBIRT principles in action. Specifically, the clinician demonstrated best practices in interviewing and communicating with a patient who was prescribed an opioid for persistent musculoskeletal pain and presented with escalating substance use.

I found the interview between the patient and healthcare provider very educational and informative. It help[ed] me think about how to ask these types of questions without offending the patient. (DNP student)

#### 5.2.3 Student Teams

A recurring theme was the impact other student team members had on the overall impressions of the activity. IPE worked best when all participants were willing to contribute. When key ‘teamwork’ features were lacking, such as group member engagement, diversity of professions represented, or group cohesion, this was apparent to participants.

##### 5.2.3.1 Group Member Engagement

The importance of active and effective collaboration surfaced in student comments (*n* = 27). When student team members actively engaged in discussions, students generally had a positive experience.

Inter‐professional team members collaborated effectively and willing[ly] in accordance to [the] patient's care. (Pharmacy student)

However, when one or more team members did not actively participate, the whole team suffered.

The majority of my group members were not very engaged in the discussion. I am not sure how that can be improved as it is dependent on the participants and their willingness to participate in the activity. (DNP student)

While our activity included a few minutes for introductions and roving facilitators, students recommended ways to increase engagement included adding an icebreaker, assigning a small group facilitator to each breakroom and assigning roles for every team member.

I think adding a simple ice breaker or allowing time to get to know some of the individuals in our small groups before getting started. For example, learning some background information like area of interest or specialization. It feels a little awkward to dive in without much prior engagement. (BASW student)

The breakout room may need a facilitator to help guide conversations. (Pharmacy student)

Having a role for every group member so everyone has to contribute something. (DNP student)

##### 5.2.3.2 Diversity of Professions Represented

While every effort was made to assign different professions to each team, the strategic assignment of diverse interprofessional groups to support collaboration was identified as key to positive interprofessional collaboration and team experience. As discussed earlier, for students who were in student teams that involved diverse professions, interprofessional student collaboration was the highlight and most valued aspect of the IPE encounter. However, when teams lacked diversity of professions, gaps were evident, that is, knowledge gaps related to creating holistic care plans. Understandably, including diverse professions on every student team was highly recommended (*n* = 9).

It should be required for more professions. I feel like most groups in the section were full of pharmacy students so it's harder to collaborate when we are all in the same field. (Pharmacy student)

…would like to have more student [variety] like Physical therapist, dietitian, nutrition etc. (Pharmacy student)

##### 5.2.3.3 Professional Respect

Professional respect was recognised as important for a positive team experience (*n* = 4). The comments below highlight contrasting student team experiences, influenced by the existence of or lack of mutual respect and professional etiquette.

Team members were already respectful and willing to work together to improve patient health outcome[s] holistically. (Pharmacy student)

Emphasizing general respect for all professions involved. Professions deserve to be there despite what they do or the level of education they receive. (Athletic Training student)

## 6 Discussion

Our qualitative study explored the perceived value and suggestions for improvement of a novel format for an IPE activity. The findings revealed both educational benefits and challenges associated with using a SP in a large group session, supported by team breakouts and a recorded patient‐provider video. Students consistently perceived this format as a meaningful IPE activity that supported their learning, particularly in navigating complex patient interactions.

Enhancing realism of IPE through simulation and SPs has been widely recognised as a strategy to improve learner engagement and skill acquisition. Nestel et al. (2017) emphasised the importance of simulation design and professional standards in SP methodology to ensure effective learning experiences. Although our follow‐up encounter used only one SP, contrasting with the initial encounter where each team interacted with its own SP for the entire session, students still valued the SP interaction. This finding aligns with Hallin et al. (2011) who found that patients perceived higher quality of care and communication when treated by interprofessional student teams, reinforcing the educational value of collaborative practice settings.

Students particularly appreciated the combination of small group discussions with their interprofessional team and large group interactions with the SP. The modelling of a challenging conversation in the video, followed by the opportunity to ask follow‐up questions during the Q&A session, supported deeper reflection and learning. This dual approach—observation and interaction—mirrors findings from Thistlethwaite, who highlighted the importance of experiential learning in developing interprofessional competencies (Thistlethwaite 2012).

Importantly, the use of a single SP per session is more cost‐effective than assigning one SP per team, making it a feasible model for programs with limited resources. This approach may also reduce stress for students navigating sensitive topics, such as SUD by allowing them to observe and reflect before engaging directly. Reeves et al. (2013) noted that learner engagement in IPE is influenced by multiple factors, including team composition and facilitator style and that simulation‐based learning can enhance attitudes and collaborative skills.

### 6.1 Strengths and Limitations of the Work

These data represent student responses from a single geographic area and thus may not be generalizable. However, a strength of this study is broad representation from eight programs and four institutions. All data are self‐reported and may include bias, for example, social desirability. Because completing the post‐session survey was optional, we may have missed valuable feedback from students who did not wish to take the time to complete it. Students were able to provide anonymous responses that did not impact course grades, thus facilitating more honest comments.

### 6.2 Recommendations for Further Research

Future studies might consider randomised designs using various IPE teaching methodologies to yield more confidence in findings about the superior learning activities. The online open‐ended questions used in our study may not have stimulated detailed responses. Interviews or focus groups might provide more in‐depth responses. Prior research suggests that communication styles differ across disciplines, and deeper exploration into the conversations occurring within the small IPE group settings could yield valuable insights for effective collaborative teamwork and decision‐making (Bouchez et al. 2024).

Additionally, comparative studies examining the effectiveness of centralised versus distributed SP models could help clarify the trade‐offs between cost, realism and learner outcomes. Investigating how different SP formats influence student engagement, empathy and retention of knowledge would be particularly valuable.

### 6.3 Implications for Policy and Practice

When choosing the timing and alignment of IPE activities for students from different health professions, programs must consider prior learning and experience and give students enough preparation for a particular case. Our professionally diverse student learners valued having curricular resources to prepare themselves for success during synchronous sessions. Our interprofessional faculty team created learning materials specifically for the longitudinal offerings and curated widely available materials. While faculty aimed to limit pre‐class work to avoid overwhelming students, students suggested additional or enhanced resources on pharmacotherapy for OUD and AUD, motivational interviewing, alternative pain management and holistic approaches for future inclusion.

Our analysis highlights the perceived importance of careful planning of student teams, focusing on team member engagement. Students reported that interprofessional team dynamics were at times greatly impacted by the composition of student teams. Similar to previously reported findings of students' experience with IPE, student willingness to collaborate effectively was essential (Peeters et al. 2024; Telford and Senior 2017). Learners perceived an enhanced experience when all team members prepared and engaged fully. Importantly, when a student did not engage fully (i.e., was unprepared or did not contribute), the learning experience was diminished for their entire interprofessional group. This experience was more detrimental when a disengaged student was the only representative of their profession on that interprofessional team, since other members of the team lost the opportunity to benefit from that profession's unique knowledge, perspective and experience.

Learners also highlighted the value of the diversity of professions represented within their teams. Historically, interprofessional team diversity has been limited by factors including program size, geographic location, scheduling constraints and physical learning space availability. Our findings reinforce the importance of creative solutions to expand inclusivity and group engagement. For example, if an IPE activity included only medicine and nursing students, a video interaction with an SP and a pharmacist modelling discussing medications could help fill gaps in professional representation.

Student feedback provided a rich, contextualised understanding of their experience in this IPE activity. Their perspectives align with other studies showing positive gains in nursing skills, confidence and/or competence using simulation case‐based scenarios for other public health priorities such as mental health (Piot et al. 2022) and delirium (Ho et al. 2021). While there is consensus that doctorally prepared nurses should be skilled in interprofessional cooperation, inconsistencies in higher education programs persist (McBride‐Henry et al. 2024). The Interprofessional Education Collaborative (IPEC) core competencies, revised in 2023, emphasise values such as cultural humility, equity and social justice (Interprofessional Education Collaborative 2023). Curricular revision to incorporate SPs and simulation methods to address these themes could improve the learning experience and better align with updated IPEC core competencies while meeting discipline‐specific educational priorities.

## 7 Conclusions

This study examined student perspectives of a virtual simulation follow‐up encounter within a longitudinal IPE case‐based training, focused on caring for a patient with chronic pain and SUD. Qualitative analysis of student perspectives on the IPE activity revealed key features of the IPE encounter that enhanced student learning including the facilitation of interprofessional student collaboration through both small and large group discussions and incorporation of relevant content with sufficient preparation materials and resources. Use of small group IP teams, a clinician‐patient interaction video paired with a large group Q and A session with a single SP per IPE session, allowed for a cost‐effective approach to enhance realism. Features that detracted from the student experience included challenges with group member engagement, and limited diversity of professions represented within student teams. The findings from this study may inform academic programs as they develop innovative IPE curricula that meet both programmatic and accreditation standards.

## Author Contributions

All authors have agreed on the final version and meet at least one of the following criteria: (1) substantial contributions to conception and design, acquisition of data or analysis and interpretation of data; (2) drafting the article or revising it critically for important intellectual content. https://www.icmje.org/recommendations/.

## Funding

The research reported in this publication was supported by the Substance Abuse and Mental Health Services Administration (Award 1H79FG000075‐01). The content is solely the responsibility of the authors and does not necessarily represent the official views of the funding agency or affiliations, including SAMHSA.

## Ethics Statement

Washington State University Human Research Protection Program certified this study as a not Human Subjects study and waived the need for Institutional Review Board approval.

## Conflicts of Interest

The authors declare no conflicts of interest.

## Data Availability

The data that support the findings of this study are available on reasonable request from the corresponding author. The data are not publicly available due to privacy restrictions.
